# Participatory, Virologic, and Wastewater Surveillance Data to Assess Underestimation of COVID-19 Incidence, Germany, 2020–2024

**DOI:** 10.3201/eid3009.240640

**Published:** 2024-09

**Authors:** Anna Loenenbach, Ann-Sophie Lehfeld, Peter Puetz, Barbara Biere, Susan Abunijela, Silke Buda, Michaela Diercke, Ralf Dürrwald, Timo Greiner, Walter Haas, Maria Helmrich, Kerstin Prahm, Jakob Schumacher, Marianne Wedde, Udo Buchholz

**Affiliations:** Authors affiliation: Robert Koch Institute, Berlin, Germany

**Keywords:** COVID-19, respiratory infections, severe acute respiratory syndrome coronavirus 2, SARS-CoV-2, SARS, coronavirus disease, zoonoses, viruses, coronavirus, incidence of notified cases, pandemic public health and social measures, participatory surveillance, wastewater surveillance, Germany

## Abstract

Using participatory, virologic, and wastewater surveillance systems, we estimated when and to what extent reported data of adult COVID-19 cases underestimated COVID-19 incidence in Germany. We also examined how case underestimation evolved over time. Our findings highlight how community-based surveillance systems can complement official notification systems for respiratory disease dynamics.

To monitor COVID-19 epidemic spread, the World Health Organization tracked worldwide incidence by relying on notification data of laboratory-confirmed cases (*1*). In Germany, public health and social measures (PHSM), such as lockdowns and testing policies, were linked to COVID-19 incidence measured by the country’s routine notifiable disease surveillance system, particularly in the first 1.5 years of the pandemic.

We examined how sensitively the national notifiable disease surveillance system reflected the true COVID-19 incidence in Germany. Our intent was to date and quantify changes in underestimation of national notifiable disease surveillance­–derived COVID-19 incidence by relating it to participatory, virologic, and wastewater surveillance systems and to identify PHSM that contributed to changes in surveillance sensitivity.

## The Study

Our indicator of interest was adult COVID-19 notification incidence in Germany, hereafter GNS-I (German notification system incidence), during 2020–2024. In the notification system, SARS-CoV-2–positive test results were notified to local health authorities, including samples taken from physician practices, citizen testing sites, and systematic testing in workplaces and schools. The system only reported PCR-positive cases; thus, non–PCR-confirmed citizen self-tests were not included in GNS-I data. 

We used 2 comparison indicators to estimate COVID-19 incidence: GrippeWeb virologic positivity rate incidence (GW-VPR-I) and GrippeWeb self-reported positivity incidence (GW-SR-I) (Table). GW-VPR-I is incidence among adults calculated through combined data from the GrippeWeb participatory surveillance system (*1*) and from virologic sentinel surveillance in primary care settings (*2*), as described previously (*3*). GW-SR-I is self-reported laboratory or self-testing results from GrippeWeb.

**Table:** List of surveillance systems and indicators used for participatory, virologic, and wastewater surveillance data to assess underestimation of COVID-19 incidence, Germany, 2020–2024*

Abbreviations	Definition	Description and formulas
Surveillance systems		
GNS	German notification system	Mandatory notification system for infectious diseases according to German Infection Protection Act
GW	GrippeWeb	Participatory ILI and non-ILI online surveillance system for the general population, which began in 2011
VSS	Virological surveillance system	Established in primary care practices
WWS	Wastewater surveillance system	Monitors aggregated SARS-CoV-2 viral load in wastewater and began during calendar week 22 2022
Indicator		
GNS-I	German notification system incidence†	COVID-19 incidence reference indicator using data from GNS
GW-VPR-I	GrippeWeb and virologic positivity rate incidence†	COVID-19 incidence comparison indicator using data from GrippeWeb and VSS
		Formula: Weekly GW-VPR-I = GW ILI incidence × VSS SARS-CoV-2 positivity rate among ILI patients + GW non-ILI incidence × VSS SARS-CoV-2 positivity rate among non-ILI patients
GW-SR-I	GrippeWeb self-reported testing results†	COVID-19 incidence comparison indicator using GrippeWeb self-reported pathogen detection results for self-tested or laboratory-confirmed positive tests; data collection started in calendar week 27, 2022
		Formula: COVID-19 incidence measured by GW-SR-I = weekly number of adult GW participants with any acute respiratory infection and a positive COVID-19 test ÷ weekly number of all reports of adults (ill or not ill)
SC2-VL-WW	Aggregated SARS-CoV-2 viral load in wastewater	COVID-19 comparison indicator using WWS system and expressed as the number of SARS-CoV-2 gene fragments per liter in wastewater
UEF	Underestimation factor	Two underestimation factors were calculated as an indicator to estimate the sensitivity of GNS-derived COVID-19 incidence with the help of GW and VSS surveillance data
		Formulas: UEF_GW-VPR-I_ = COVID-19 incidence measured by GW-VPR-I ÷ COVID-19 incidence measured by GNS-I UEF_GW-SR-I_ = COVID-19 incidence measured by GW-SR-I ÷ COVID-19 incidence measured by GNS-I

*ILI, influenza-like illness. †COVID-19 incidence indicator.

Each week, ≈8,000 GrippeWeb participants in Germany self-report symptoms related to any kind of acute respiratory illness (ARI), which includes any illness with sore throat, cough, or fever. Participants also report potential test results. ARI are dichotomized into influenza-like illness (ILI; i.e., fever with sore throat or cough) and non-ILI. GrippeWeb provides ARI, ILI, and non-ILI incidence rates in the general population (*1*). The National Influenza Centre conducts virologic surveillance in cooperation with ≈140 practices (general and pediatric practices) that submit nasal or throat swab samples from ARI patients (*4*). Samples are analyzed by real-time PCR for different respiratory pathogens, including SARS-CoV-2. 

To compare GNS-I with GW-VPR-I, we used incidence from calendar week (CW) 40 of 2020 through CW 4 of 2024 (CW40/2020–CW04/2024). To compare GNS-I with GW-SR-I, we included CW27/2022 (beginning of collection of self-reported SARS-CoV-2 detections in GrippeWeb) through CW04/2024. We smoothed GW-VPR-I and GW-SR-I data by using the locally estimated scatterplot smoothing (LOESS) method (*5*).

Beginning in CW22/2022, SARS-CoV-2 was monitored weekly by wastewater surveillance (WWS) in <153 wastewater treatment plants (*6*). Data were aggregated as SARS-CoV-2 viral load in wastewater (SC2-VL-WW). We also used LOESS to smooth weekly mean SC2-VL-WW data.

We used an underestimation factor (UEF) to express sensitivity of GNS-I by GW-VPR-I (UEF_GW-VPR-I_) and GW-SR-I (UEF_GW-SR-I_), which we calculated as the weekly ratio of smoothed GW-VPR-I and GW-SR-I relative to nonsmoothed GNS-I (Table; Figure). In addition, we gathered information on dates of pandemic related PHSM.

**Figure:**
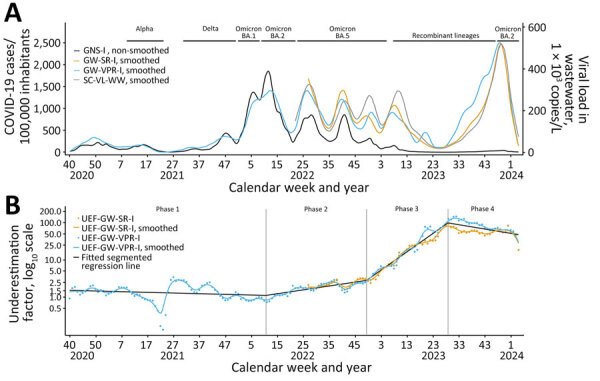
Incidence and underestimation factors in a study of participatory, virologic, and wastewater surveillance data to assess underestimation of COVID-19 incidence, Germany, 2020–2024. A) Smoothed and unsmoothed surveillance data on COVID-19 incidence (cases/100,000 adult population) compared with wastewater viral load. SARS-CoV-2 variant phases in Germany are labeled. B) Two different UEFs plus common piecewise trendline of smoothed UEF and timeframes for phases calendar week 40 of 2020 through calendar week 4 of 2024. Vertical lines mark the breakpoints between COVID-19 phases with different degrees of underestimation. GNS-I, incidence from German notification system; GW-SR-I, GrippeWeb self-reported incidence; GW-VPR-I, GrippeWeb and virologic positivity rate incidence; SC2-VL-WW, aggregated SARS-CoV-2 viral load in wastewater; UEF, underestimation factor.

In general, measured COVID-19 incidence by GNS-I, GW-VPR-I, and GW-SR-I, as well as SC2-VL-WW, all agreed in timing of COVID-19 waves (Figure, panel A). GW-VPR-I was similar to the GNS-I until ≈CW17/2022, after which the 2 curves diverged. From CW27/2022, GW-SR-I aligned with GW-VPR-I. SC2-VL-WW confirmed the course of GW-VPR-I and GW-SR-I, which indicated that COVID-19 waves that peaked during CW26/2022, CW38/2022, and CW50/2022 were substantially stronger than suggested by GNS-I.

We identified 4 major sensitivity phases of GNS-I and estimated a segmented linear regression to specify 3 breakpoints (*7*,*8*). We calculated a common piecewise trendline of the smoothed UEF_GW-VPR-I_ and UEF_GW-SR-I_ data (Figure, panel B). During phase 1, CW40/2020–CW10/2022, the linear trend of UEF_GW-VPR-I_ varied ≈1.1–1.5, indicating close agreement between GNS-I and GW-VPR-I. Two COVID-19 waves, driven by Omicron BA.1, peaking in CW05/2022, and BA.2, peaking in CW11/2022, were still well captured by GNS-I. During that time, many workplaces, hospitals, nursing homes, kindergartens, and schools tested regularly for SARS-CoV-2. However, during CW10/2022–CW17/2022, regular testing at workplaces and schools was gradually discontinued. Until the end of phase 2 (CW49/2022), smoothed GW-VPR-I and GW-SR-I slowly increased to ≈2.8 (UEF_GW-VPR-I_ was 2.7; UEF_GW-SR-I_ was 2.9). 

At the end of 2022, no-cost testing ceased for all citizens, after which we noted a steep increase of both smoothed UEFs during phase 3 (CW49/2022–CW28/2023): UEF_GW-VPR-I_ increased from ≈2.7 to 110.1 and UEF_GW-SR-I_ increased from 2.9 to 81.6 (Figure, panel B). SC2-VL-WW showed similar trends during that period, but GNS-I data barely captured the phase 3 waves.

Through a trend change in both UEFs, we identified a fourth phase starting around CW28/2023 that was not accompanied by PHSM changes. Smoothed UEF_GW-VPR-I_ peaked around CW32/2023, then decreased to ≈30.3; UEF_GW-SR-I_ peaked around CW28/2023, after which it fluctuated between ≈50–70. SC2-VL-WW followed the steady rise of the 2 GrippeWeb indicators and peaked in CW50/2023. GNS-I remained low in phase 4.

One limitation of our study is the incongruence among the indicators; GNS-I includes data for illnesses and asymptomatic infections, whereas GW-VPR-I and GW-SR-I only estimate illness incidence. However, because the information on presence or absence of symptoms is not always available in GNS-I data, deriving a pure COVID-19 incidence from GNS-I is not possible. Another limitation is that WWS provides viral load per liter from all population age groups, and neither incidence nor prevalence data are collected; whether the shedding properties of variants differ enough to substantially modify the viral load detected in wastewater is unknown. Last, the association of sensitivity phases and PHSM is only descriptive and ecologic in nature.

## Conclusions

Assessing the timing and degree of COVID-19 underestimation is crucial for interpretating notification system data. Until the first half of 2022, serosurveys among blood donors in Germany estimated the degree of underestimation at ≈1.5 of GNS-I, comparing well with the common piecewise trendline of UEF_GW-VPR-I_ in the same timeframe (UEF_GW-SR-I_ started from CW27/2022) (*9*). Other than cross-sectional serosurveys (*9*–*11*), approaches to estimate underestimation included analysis of fatality rates and death tolls (*12*,*13*), and a multiplier model that used reported laboratory-confirmed data as a starting point (*14*). However, none of those approaches compared weekly notification rates and, thus, cannot pinpoint sensitivity breakpoints. We compared weekly national notifiable COVID-19 incidence to 2 independent indicators estimating population-level incidence, and our findings are supported by WWS results.

We identified 2 major sensitivity breakpoints, demonstrating that PHSM introductions or cessations might have directly affected the changing sensitivity of notification data. Ending systematic testing in workplaces and schools (first breakpoint) and ending no-cost testing (second breakpoint) likely contributed to the decrease of national notifiable disease surveillance system sensitivity. The close agreement between WWS and GrippeWeb-derived incidence indicators suggests that SARS-CoV-2 wastewater data are useful for indicating trends in infection waves.

Although population-level immunity could influence the probability of persons testing COVID-19–positive to some degree, immunity mainly protects against severe disease but does not necessarily prevent infection or illness. For example, the high estimated COVID-19 incidence at the end of 2023 had weekly incidences of >2% (Figure).

As Germany transitioned from the pandemic to endemic phase and implemented a stepwise reduction in testing, GNS-I became less capable of reflecting actual COVID-19 incidence. Our study results stress the value of additional community-based and wastewater surveillance systems to complement official notification systems (*15*). Community-based surveillance can describe the epidemiologic situation, particularly when PHSM, such as testing policies, are lifted and testing access decreases. Thus, systems like GrippeWeb (and wastewater surveillance) will be increasingly crucial, especially for respiratory diseases of epidemic and pandemic potential.
